# Carbohydrate PEGylation, an approach to improve pharmacological potency

**DOI:** 10.3762/bjoc.10.147

**Published:** 2014-06-25

**Authors:** M Eugenia Giorgi, Rosalía Agusti, Rosa M de Lederkremer

**Affiliations:** 1 CIHIDECAR-CONICET, Departamento de Química Orgánica, Facultad de Ciencias Exactas y Naturales, Universidad de Buenos Aires, Pabellón II, Ciudad Universitaria, 1428 Buenos Aires, Argentina

**Keywords:** bioavailability, carbohydrates, conjugates, glycoPEGylation, multivalent glycosystems, multivalent PEGylation

## Abstract

Conjugation with polyethylene glycol (PEG), known as PEGylation, has been widely used to improve the bioavailability of proteins and low molecular weight drugs. The covalent conjugation of PEG to the carbohydrate moiety of a protein has been mainly used to enhance the pharmacokinetic properties of the attached protein while yielding a more defined product. Thus, glycoPEGylation was successfully applied to the introduction of a PEGylated sialic acid to a preexisting or enzymatically linked glycan in a protein. Carbohydrates are now recognized as playing an important role in host–pathogen interactions in protozoal, bacterial and viral infections and are consequently candidates for chemotherapy. The short in vivo half-life of low molecular weight glycans hampered their use but methods for the covalent attachment of PEG have been less exploited. In this review, information on the preparation and application of PEG-carbohydrates, in particular multiarm PEGylation, is presented.

## Introduction

In recent years, the modification of biotherapeutics by covalent conjugation with polyethyleneglycol (PEG) known as PEGylation has emerged as an effective strategy to improve the therapeutic potential of drugs through less frequent dosing [1–3]. PEG is a biologically inert, non-immnunogenic linear polyether diol that confers proteins greater solubility in aqueous and organic media. It is being used in pharmaceutical areas not only to enhance water solubility and reduce immunogenicity but also to increase in vivo circulation half-life by preventing enzymatic degradation and renal clearance [4]. Numerous examples of bioconjugation with PEG have been reported including, among others, proteins located in adenovirus coat for vaccine development [5], antibodies or antibody fragments to prolong their circulating half-lives in vivo [6] and selective alkylation and acylation of amino groups in a somatostatin analog using two different PEG reagents [7]. Also, PEGylation of low molecular weight drugs in order to increase solubility [8], prolong the in vivo action [9] or for targeting drug delivery [10] has been described. The potent anti-inflammatory drug dexamethasone was coupled to a multifunctional PEG, prepared by a click reaction, for treatment of rheumatoid arthritis [11]. A heterobifunctional PEG has been conjugated with both paclitaxel, a potent anticancer drug, and alendronate, a bone-targeting biphosphonate, in order to obtain strong bone tropism and fast drug release [12]. An enzymatic method using a microbial transglutaminase was described for PEGylation of human growth hormone [13].

Glycans have been recognized as immunodominant epitopes in antigenic glycoconjugates [14]. Carbohydrates participate in molecular recognition events such as host–pathogen interactions, responsible for mammal infections, and are candidates for chemotherapy [15]. Moreover, synthesis of multivalent carbohydrate ligands provide higher affinity for receptors as described for Shiga-like toxins [16] and other systems [17].

Several excellent reviews have been published on PEGylation of proteins, including PEG-drugs in the market [2,18–21]. However, PEGylation of carbohydrate molecules has been less exploited and studies have been focused on polysaccharides or on carbohydrates linked to proteins. A review dedicated to PEGylated chitosan derivatives has been published [22].

In the present review we present different approaches used for modification of glycans by covalent conjugation with PEG reagents, in particular with multiarm PEGs, with the aim to increase the loading of the active sugar. Multivalent glycomolecules have proven to mediate or inhibit a variety of biological or pathological processes [17,23].

## Review

### Polyethylene glycol (PEG) derivatives

**Figure 1:**
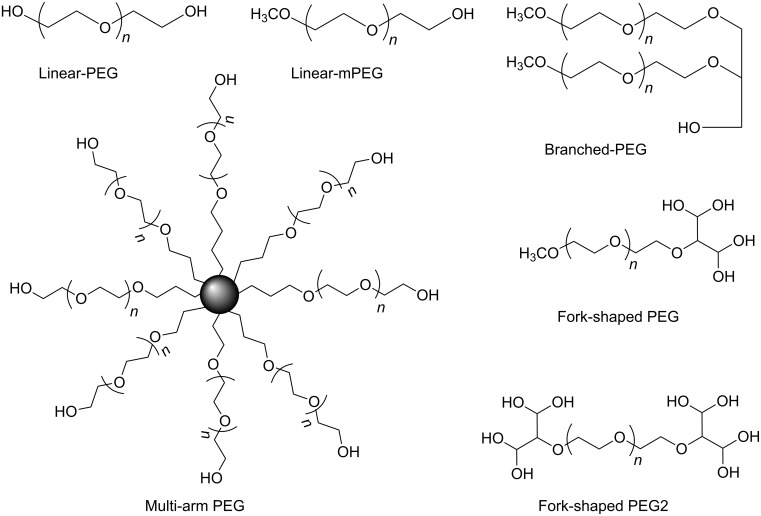
Types of PEG utilized for derivatization of drugs and peptides.

**Figure 2:**
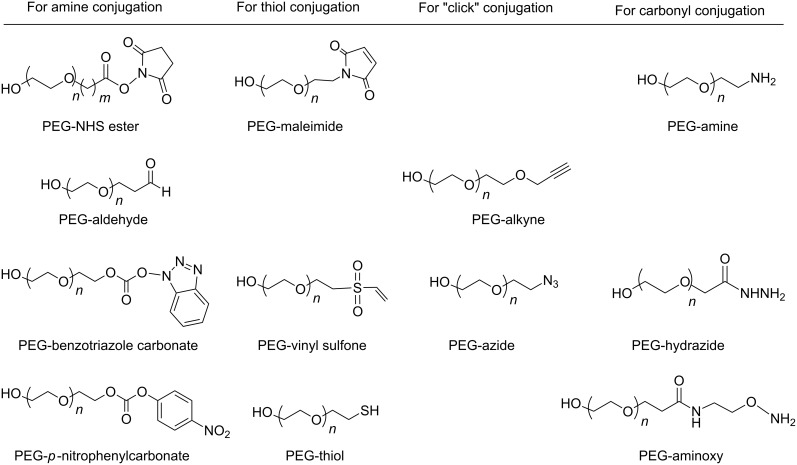
Activated PEG derivatives for conjugation.

Polyethyleneglycol is an amphiphilic polymer consisting of repeating units of ethylene oxide which may be assembled in linear or branched structures to give a range of PEGs with different shapes and molecular weights (Figure 1). PEG must be activated for further conjugation by substitution of terminal OH by a functional group that could react with an appropriate site in the molecule to be conjugated, maintaining its biological activity. Examples of activated PEGs are shown in Figure 2. Multiarm PEGs have the advantage of presenting several sites for conjugation and, in the higher MW conjugates, the arms are far away enough from each other to allow independent interaction with the target site.

### GlycoPEGylation of proteins

PEGylation of proteins is usually performed on the ε-amine group of lysine or on the unprotected α-amino of the *N*-terminal amino acid using *N*-hydroxysuccinimidyl (NHS) activated PEGs or aldehyde PEGs. This conjugation leads to heterogeneous products, depending on the number of lysine residues in the molecule. Random PEGylation may have undesired steric effects, shielding active sites in the protein or disrupting its tertiary structure [24].

PEGylation may be also directed to the side-chain amide nitrogen of Asn. In order to improve the pharmacokinetic properties of a protein, *N*-PEGylation may be used additively to *N*-glycosylation since both modifications stabilize the protein by different mechanisms [25]. Also, glutamine residues on intact or chimeric proteins can be combined with alkylamino-PEG derivatives by the use of a transglutaminase [26].

Several methods have been developed for site-directed PEGylation. One of the most popular involves the reaction of the thiol group in one or two cysteine residues with appropriate PEG derivatives. The cysteine could be originally present in the protein or introduced by mutagenesis [27–28]. The C-terminus of the human growth hormone was PEGylated using a two-step strategy in which a linker was first incorporated by a carboxypeptidase-catalyzed transpeptidation and then used for the ligation of the PEG moiety [29]. A more specific and irreversible attachment of a single PEG molecule has been achieved by the use of a [3 + 2] cycloaddition reaction of an alkyne-bearing PEG reagent and an azide-functionalized tyrosine residue genetically incorporated on human superoxide dismutase-1 [30].

GlycoPEGylation, targeting carbohydrate sites, was conceived to produce a more homogeneous product with lower steric effects [31]. The strategy is based on the finding that certain PEGylated nucleotide-sugars are effectively transferred to a glycan acceptor by the corresponding glycosyltransferase.

**Scheme 1:**
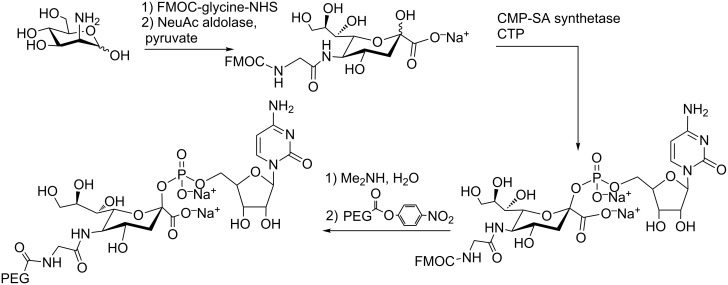
Chemoenzymatic method for the preparation of PEG-CMP-SA, adapted from [32–33].

A modified sialic acid PEGylated at the 5’-amino position in the CMP nucleotide (CMP-SA-5-NHCOCH_2_NHPEG) can be transferred to a glycan acceptor in a glycoprotein by a sialyltransferase [32–33]. A chemoenzymatic method for its preparation is shown in Scheme 1. It is based on the coupling of Fmoc-glycyl-mannosamine with pyruvate catalized by SA-aldolase to afford the *N*-protected sialic acid. After reaction with CTP catalyzed by CMP-sialic acid synthetase, the nucleotide is deprotected and the free amine is utilized as a locus to PEG attachment.

**Scheme 2:**
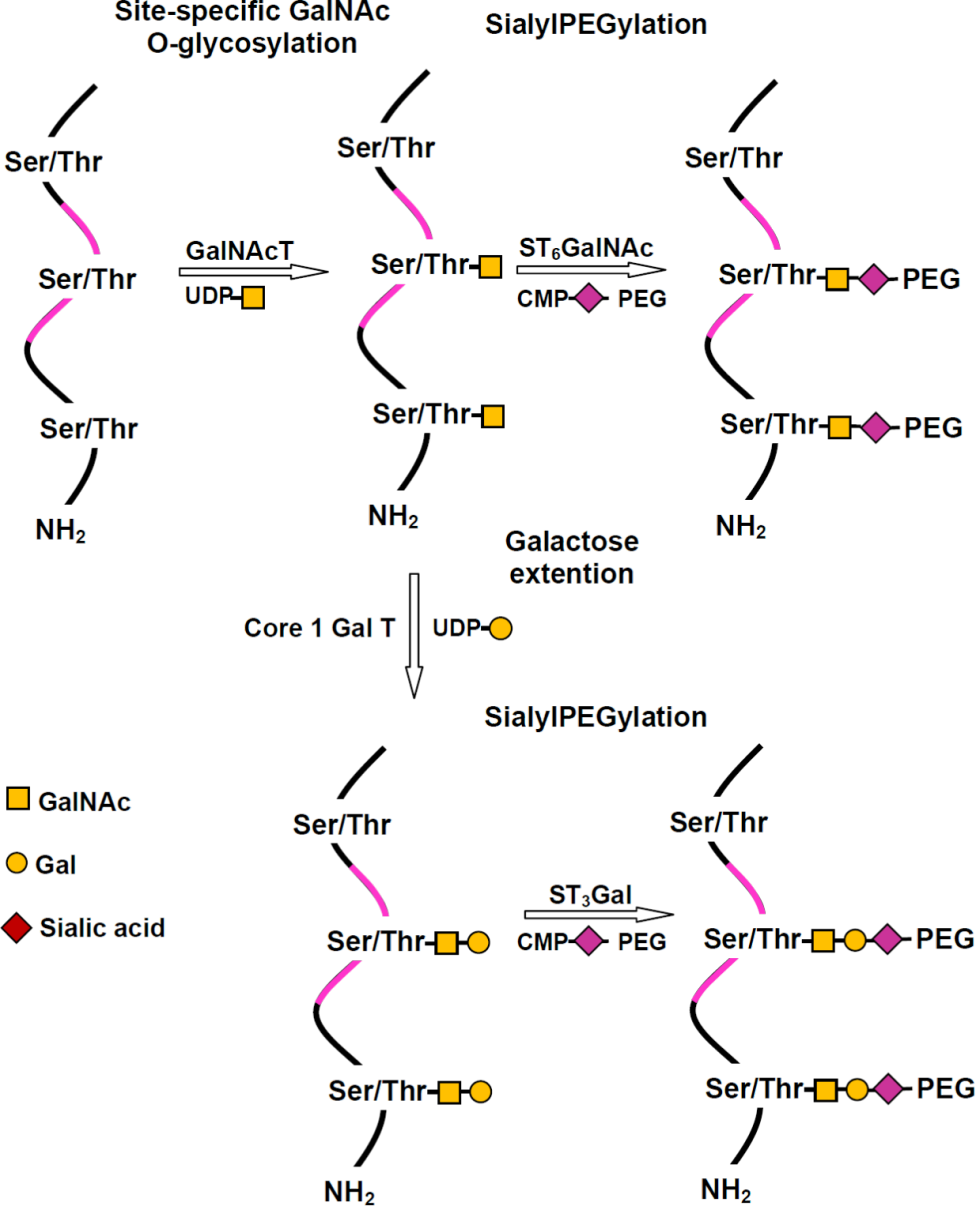
GlycoPEGylation by sequential in vitro, enzyme mediated, *O*-glycosylation followed by transfer of PEGylated sialic acid, adapted from [31].

The introduction of the PEGylated sialic acid into the glycoprotein takes place in two steps. First, an *O*-glycan is introduced enzymatically and second, PEGylated sialic acid is transferred to the glycan by a sialyltransferase. The serine or threonine residues in the *O*-glycosylation sites serve as acceptors for GalNAc using a convenient GalNAc transferase. This unit can be galactosylated by a galactosyltransferase and both, the monosaccharide and the disaccharide, may be acceptors for PEG-sialic acid (Scheme 2). This technique was applied to polypeptides used clinically and has the advantage that it is easier to produce a recombinant protein using *E. coli* than to obtain the glycosylated forms in eukaryotic cells [31].

### Chemical glycation of a protein and PEGylation after periodate oxidation

**Scheme 3:**
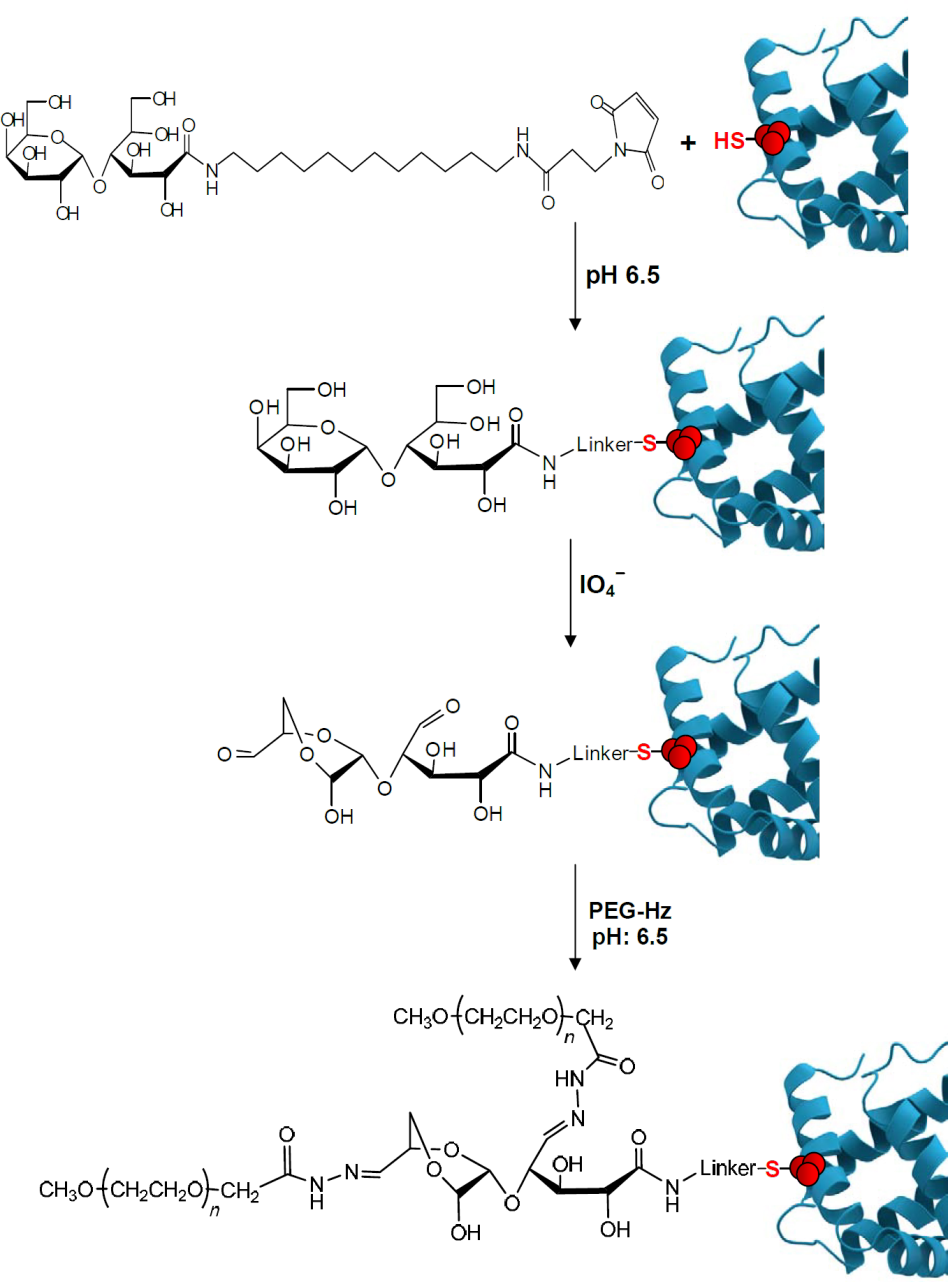
Chemical glycation of a protein and PEGylation after periodate oxidation, adapted from [34].

A small glycan may be also introduced by chemical ligation to an inaccessible aminoacid in a natural protein, like Cys^34^ in human serum albumin. The glycan may be oxidized by periodate to afford aldehyde groups for selective multiple coupling with a PEG hydrazide (PEG-Hz), as shown in Scheme 3 [34]. Analysis of the PEGylated species showed more than 90% conversion, whereas less than 30% of the protein was PEGylated by direct conjugation of the albumin with commercial PEG-maleimide. The PEG-Hz may undergo pH controlled hydrolysis which also depends on the number of units in the linked sugar. Therefore release of the active protein may be controlled by the structure of the sugar linker.

### PEGylation of native glycosylated proteins

PEGylation of native glycoproteins may be performed by enzymatic or chemical modification of the glycan.

#### a) Enzymatic modification of the glycan

Enzymatic PEGylation of a glycoprotein can be performed in three steps. First, the sialic acid is removed from the native protein with a sialidase and subsequently Sia-PEG is transfered to the uncovered terminal Gal units of the linked glycan taking advantage of the substrate promiscuity of the sialyltransferase ST3GalIII [35–36]. The reaction is kinetically controlled and the number of PEGs added depends on the reaction time. Finally, the unreacted galactose residues should be blocked with sialic acid to avoid hepatic clearance by the asialoglycoprotein receptor. More recently, the same group modified genetically the coagulation factor VIII used to treat Hemophilia A, in order to obtain a unique *O*-linked glycan for selective modification with PEGylated sialic acid [37].

**Scheme 4:**
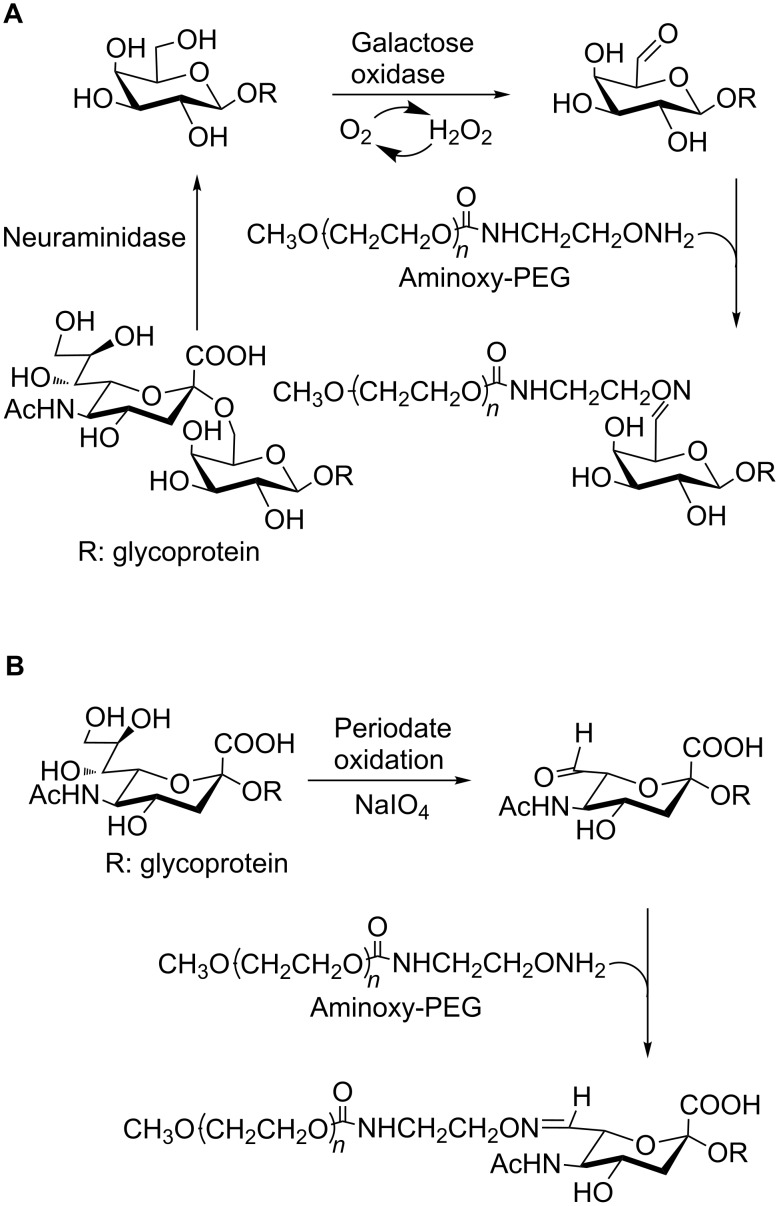
PEGylation of native glycosylated proteins after modification of the glycan. (A) Enzymatic modification of the glycan; (B) Chemical modification of the glycan, adapted from [38].

Alternatively, a terminal galactose may be oxidized at C-6 with galactose oxidase to create the reactive site in the glycan that could react with an activated PEG (Scheme 4A). As galactose is usually substituted with sialic acid, the latter procedure was applied before or after sialidase treatment [38].

#### b) PEGylation after chemical modification of the sugar chain of a glycoprotein

In this approach, a reactive group is created in the sugar of an *O*- or *N*-linked glycan by a chemical modification. The terminal residue of the *N-*linked carbohydrate in ricin A-chain has been PEGylated by mild oxidation with periodate followed by reaction with hydrazide-derivatized PEG [39]. The carbohydrate-specifically modified ricin showed better pharmacokinetic properties than the peptide amino-PEGylated or the unmodified ricin. The same technique was applied to glucose oxidase (GOx), a glycosylated dimeric protein. In this case the hydrazone was further stabilized by reduction with cyanoborohydride to afford a bioconjugate with retention of its activity as a biosensor of glucose [40]. A similar strategy was applied to the recombinant human thyroid-stimulating hormone (rhTSH, Thyrogen). Terminal sialic acids were oxidized with sodium periodate to generate aldehydes, which reacted with aminoxi-PEGs (Scheme 4B). The use of this PEGylating agent, instead of hydrazide-PEGs, generated a more stable oxime linkage with the carbohydrate aldehydes. Similar to the other gonadotropins, TSH is a glycosylated protein, and the role of the *N*-linked oligosaccharides is well established. The effect of PEG size and mono- vs multi-PEGylation was compared both in vitro and in vivo. The best performing of the products, a 40-kDa mono-PEGylated sialic acid-mediated conjugate, exhibited a 5-fold lower affinity which was however compensated by a 23-fold increase of circulation half-life [38].

**Scheme 5:**
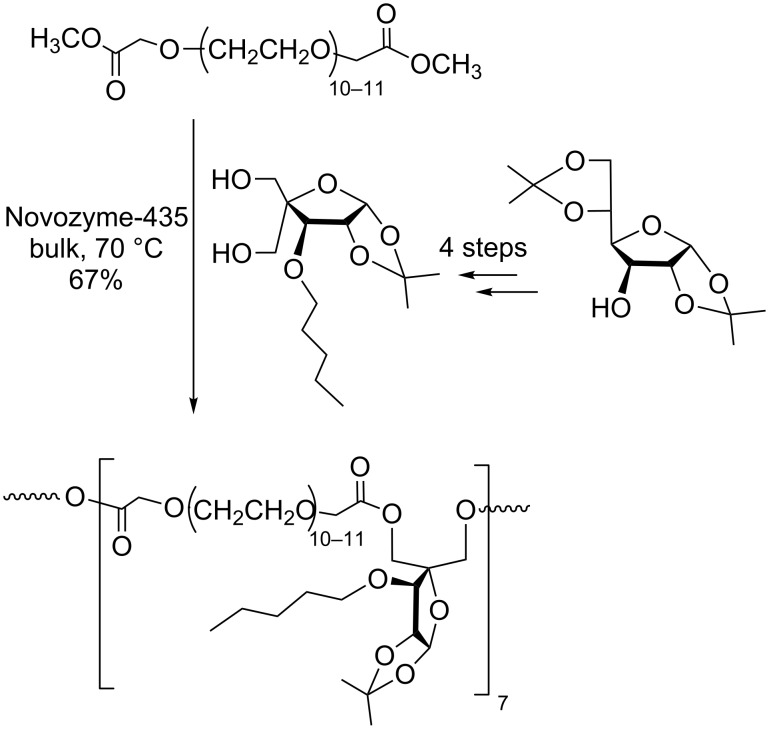
PEGylation of a pentofuranose derivative, adapted from [41].

**PEGylation of low-molecular weight carbohydrates:** Enzymatic esterification of two hydroxy methylene groups present in pentofuranose derivatives with a PEG dimethyl ester yielded sugar-PEG copolymers used for drug encapsulation. The carbohydrate monomer was obtained by a multistep synthesis starting from the easily available diacetone glucose (Scheme 5) [41].

**Scheme 6:**
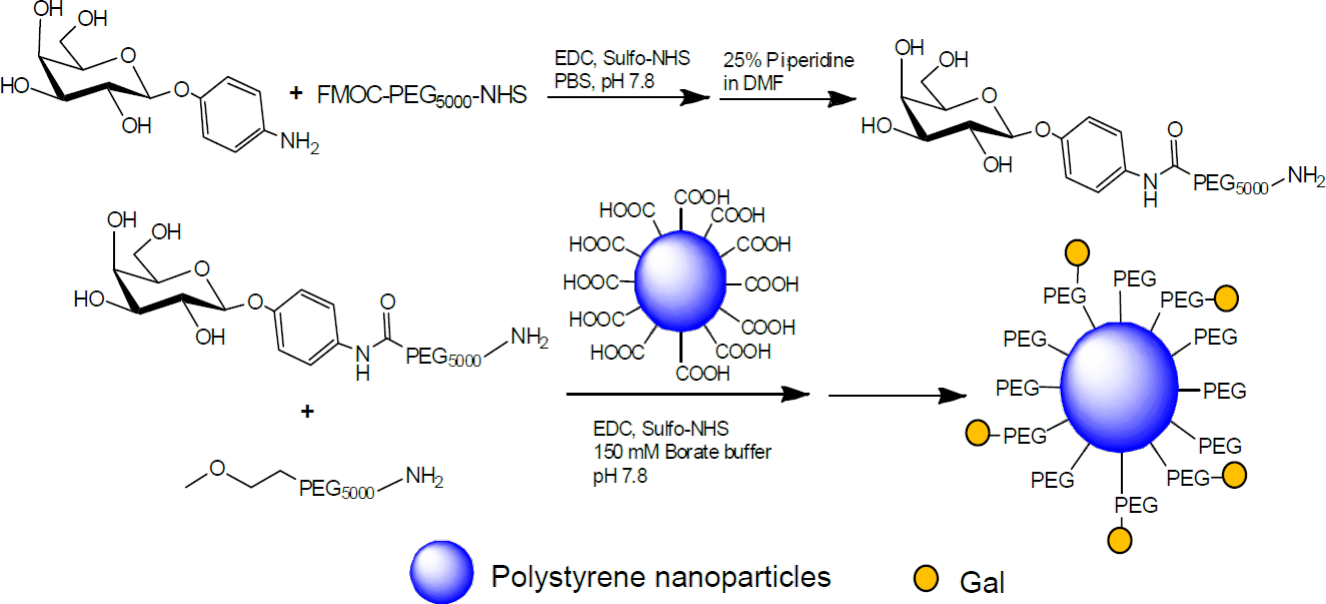
Galactosyl PEGylation of polystyrene nanoparticles, adapted from [42].

Galactose has been PEGylated and introduced in the surface of polystyrene nanoparticles in order to increase the interaction with galactose receptors. *p*-Aminophenyl β-D-galactopyranoside was coupled with a bifunctional PEG activated on one end with NHS for the combination with the aniline and a FMOC-protected amino group on the other end. After deprotection, the amine reacted with the carboxylic groups on the surface of the nanoparticles (Scheme 6) [42]. A similar approach was developed recently using poly(amidoamine) dendrimers for selective delivery of chemotherapeutic agents into hepatic cancer cells [43].

**Figure 3:**
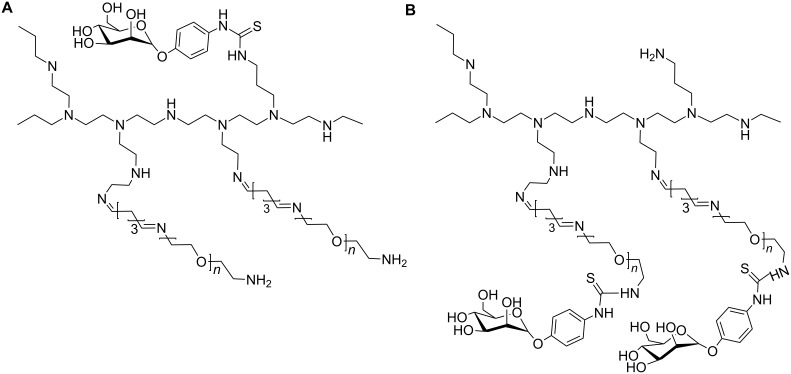
Mannosyl PEGylated polyethylenimine for delivery systems. (A) Mannose and PEG are independently linked to the PEI backbone; (B) Mannose is attached to PEI via a PEG chain, adapted from [44].

Mannose was also PEGylated in order to target drugs specifically to mannose receptors present in liver endothelial cells. Mannosyl PEGylated polyethylenimine (PEI) conjugates were synthesized either by direct coupling the mannose and the PEG chain to the PEI backbone (Figure 3A) or by attaching the mannose to PEI via a PEG chain spacer (Figure 3B). This system was used to deliver small interfering RNA (siRNA) into a murine macrophage cell line [44].

**Figure 4:**
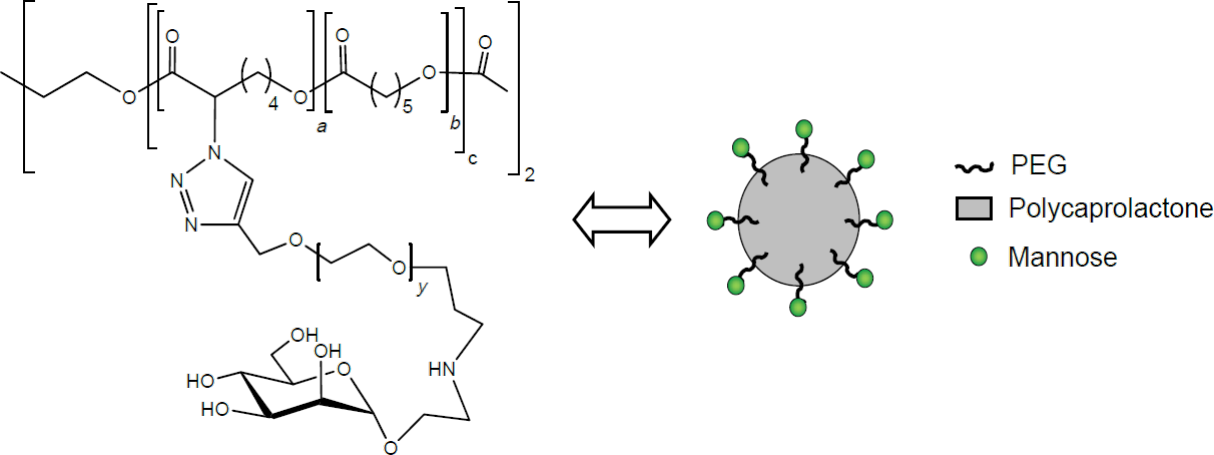
PEGylated mannose derivatives, adapted from [45].

Mannose residues as their 2-aminoethyl glycosides were attached by reductive amination to the surface of copolymer micelles of PEG with poly-ε-caprolactone for targeting dendritic cells and macrophages (Figure 4) [45]

Both mannose and galactose were attached to PEGylated nanoparticles by click-chemistry between their propargyl glycosides and a gold nanoparticles derivatized with an azide-functionalized PEG [46]. Also, several unprotected carbohydrate units of mannose, fucose or lactose, have been incorporated into the surface of PEGylated dendritic polymers by means of click chemistry. The larger dendrimer generations have demonstrated an increased capacity to aggregate lectins [47].

**Scheme 7:**
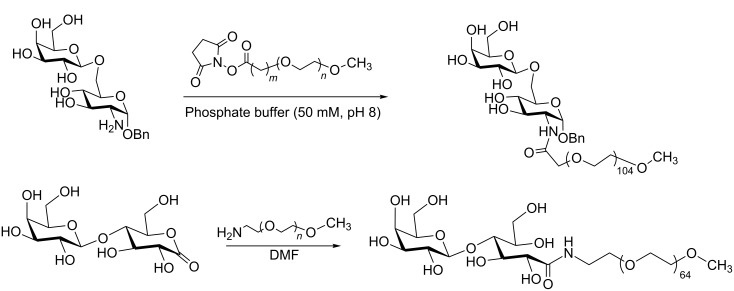
PEGylation of lactose analogs [53].

Analogs of lactose have been reported as inhibitors of the enzyme trans-sialidase (TcTS) [48], a virulence factor of *Trypanosoma cruzi* [49–51]. It was shown that lactitol prevented apoptosis caused by TcTS although it is rapidly eliminated from the circulatory system [52]. With the aim to improve bioavailability, PEGylation of lactose analogs was performed using two approaches, both depending on the formation of an amide bond. In one case the amino group was provided by the sugar and the carboxylic acid by a NHS-activated PEG and in the other approach an amino-functionalized PEG reacted with lactobionolactone (Scheme 7) [53].

**Scheme 8:**
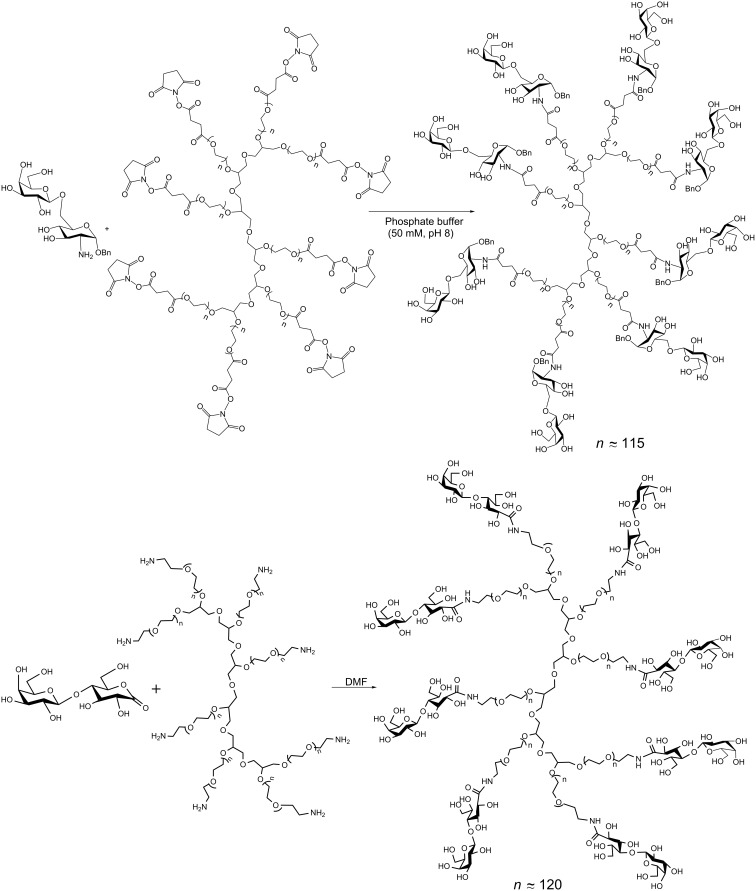
Conjugation of lactose analogs with dendritic PEGs [54].

Using linear PEGs of MW 5000 Da, no enhancement in the permanence in blood was observed. However, improved biovailability with retention of inhibition of TcTS was achieved by PEGylation with multiarm PEGs of MW 40000 (Scheme 8) [54]. In these complex conjugates, the degree of substitution is determined by ^1^H NMR spectroscopy. The identification of signals that disappear or are shifted when conjugation takes place, together with the appearance of new signals due to the sugar in well-separated regions of the spectrum are used to confirm the extent of derivatization of the multiarm PEGs.

**PEGylation of polysaccharides:** PEGylation of chitosan and chitosan derivatives for pharmaceutical applications was described [22]. Chitosan is the polysaccharide obtained from the abundant chitin by alkali or enzymatic degradation. It consists of a backbone of β-(1→4)-linked D-glucosamine units with a variable degree of *N*-acetylation. The protonated amino groups of chitosan favor interaction with negatively charged cellular surfaces. The amino groups of chitosan may be derivatized with PEG chains, thus modifying the physicochemical properties. Chitosan was first modified in the amino group of the glucosamine units with a PEG-aldehyde to yield an imine (Schiff base), which was subsequently reduced to PEG-g-chitosan with sodium cyanoborohydride [55], allowing retention of net charge. PEGylation can also be accomplished by condensation of the free amino groups with activated PEGs, such as PEG-NHS or PEG-*p*-nitrophenyl carbonate, converting the protonable amines into neutral amide or carbamate linkers.

Even though PEGylation of chitosan via the amino group is the most commonly used method a number of examples of polysaccharide derivatisation on the hydroxy groups have been reported. Chitosan-*O*-poly(ethylene glycol) graft copolymers were synthesized from *N-*phthaloylchitosan by etherification with poly(ethylene glycol) monomethyl ether (mPEG) iodide obtaining different degrees of *O*-substitution [56]. Several strategies were designed to obtain regioselective PEGylation at C-6 of the glucosamine unit [57]. Other methods of PEGylation included, among others, free radical polymerization of C-6 of the glucosamine residues with poly(ethylenglycol) acrylate [58]; free-radical polymerization of C-1 of glucosamine with mPEG [59] and 1,3-dipolar cycloaddition between the azide of an *N*-azidated chitosan and mPEG derivatives containing a triazolyl moiety [60].

**Figure 5:**
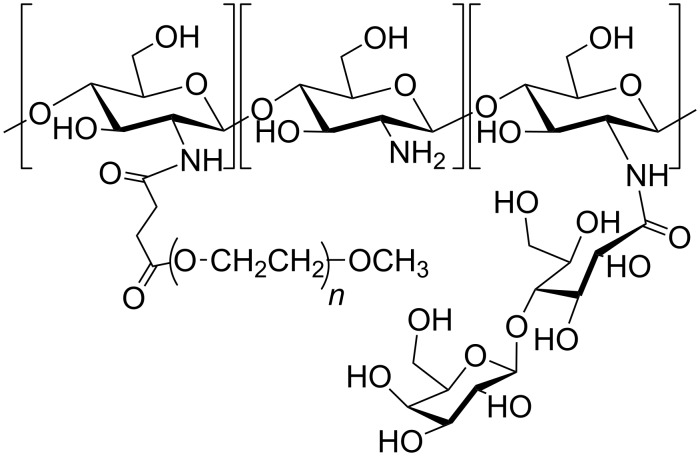
PEGylated chitosan derivative, adapted from [61].

Chitosan, partially substituted with lactobionic acid, bearing a galactose, provides a ligand for the asialoglycoprotein receptor of liver cells. Lactobionic acid formed an amide bond with the glucosamine residue, and the non-substituded amino groups of the galactosylated chitosan (GC) were further coupled with activated hydrophilic PEG to enhance its stability (Figure 5) [61].

**Figure 6:**
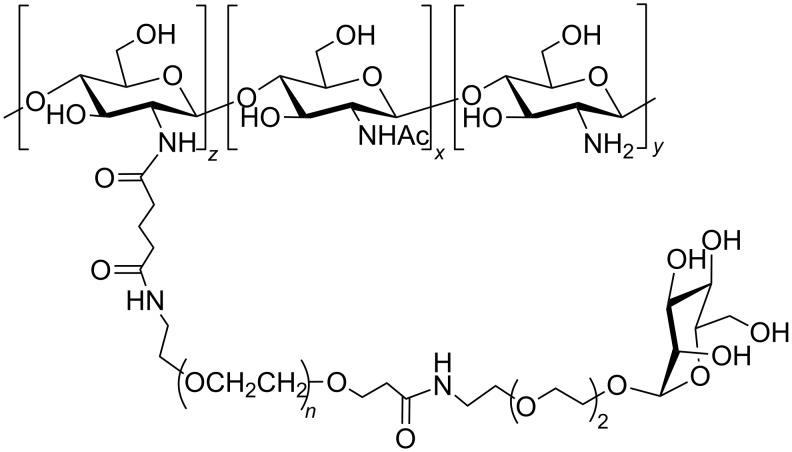
Chitosan/PEG functionalized with a mannose at the distal end, adapted from [62].

Bifunctional PEGs were used to introduce a bioactive molecule, for instance biotine, coumarin, cholesterol or mannose into the distal end of a PEG-chitosan complex (Figure 6) [62].

Fructans have been PEGylated by reaction of hydroxy-activated polysaccharides with amino-terminated methoxy PEGs. The reaction was applied to inulin [63] and to a polysaccharide from Radix Ophiopogonis [64] for improving their pharmacokinetic properties. A similar activation of the sugar has been previously applied to a dextran for further PEGylation. Hydrogels with supramolecular structures have been obtained by inclusion complexation of the PEG grafted dextrans with α-cyclodextrins. The unique thermoreversible sol-transition properties of the gels were considered interesting for drug delivery applications [65].

## Conclusion

The advantage of PEGylation of glycan structures attached to proteins is the possibility to restrict the reaction to the glycosylated site affording a product with the benefits that PEGylation can impart without the loss of activity due to random multistep PEGylation of proteins. The examples presented in this review on the PEGylation of carbohydrates show improvement of some properties such as bioavailability of drugs, in particular enzyme inhibitors, or creation of polymers with encapsulating properties for drugs. Apparently, the benefits of PEGylation were yet not extended to carbohydrate based drugs in the market. In particular, multiarm PEGylation with more available sites for glycan linking can be exploited for improvement of interaction of carbohydrates with cell receptors. We hope that this review on sugar PEGylation will provoke further studies on the subject.
